# Green Synthesis of MOF‐Based Materials for Electrochemical Reduction of Carbon Dioxide

**DOI:** 10.1002/cssc.202400684

**Published:** 2024-10-04

**Authors:** Mitra Bagheri, Mirtha A. O. Lourenço, Julien K. Dangbegnon, Nicolò B. D. Monti, Luís Mafra, Fabrizio Pirri, Juqin Zeng

**Affiliations:** ^1^ Istituto Italiano di Tecnologia – IIT Centre for Sustainable Future Technologies (CSFT) Via Livorno 60 Turin 10144 Italy; ^2^ Department of Applied Science and Technology (DISAT) Politecnico di Torino Corso Duca degli Abruzzi 24 Turin 10129 Italy; ^3^ CICECO – Aveiro Institute of Materials Department of Chemistry University of Aveiro 3810–193 Aveiro Portugal

**Keywords:** Carbon dioxide, Electrocatalysis, Zeolitic imidazolate framework, Steam-assisted dry gel method, Metal center

## Abstract

Porous ZIF‐8 and ZIF‐67 were synthesized via a green steam‐assisted dry‐gel technique and investigated as potential catalysts for CO_2_ electroreduction. The synthesis conditions are found to significantly influence the growth of these metal‐organic frameworks (MOFs). Notably, the water content employed during synthesis plays a crucial role in shaping the morphological properties of ZIF‐8. Specifically, a moderate water content results in the formation of uniform ZIF‐8 with a size distribution ranging from 240–440 nm. During CO_2_ electroreduction, these morphological properties exert substantial effects on the selectivity for CO formation, thereby facilitating the production of syngas with adjustable CO: H_2_ ratios. This feature holds promise for the widespread adoption of syngas as a clean alternative to fossil fuels, offering potential benefits for electricity generation and liquid fuel production. Despite sharing similar structural properties with ZIF‐8, ZIF‐67 exhibits distinct performance characterized by its limited selectivity for CO_2_ electroreduction. This discrepancy is attributed to the different metal centers of the two MOFs, resulting in the distinct activation of CO_2_ and H_2_O molecules and their further reduction. This finding highlights the critical role of metal centers in MOF‐based materials for electrocatalysis application.

## Graphical abstract

This work explores the potentiality of metal organic frameworks as electrocatalysts for the CO_2_ reduction reaction (CO_2_RR). Different sized ZIF‐8 and its isostructural ZIF‐67 were successfully synthesized via a green steam‐assisted route. The comprehensive study shows that both particle size and metal center affect the catalytic performance for CO_2_RR, with the latter playing the central role.

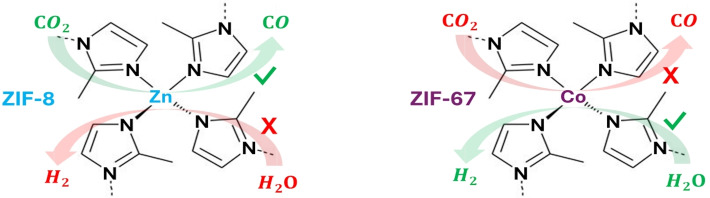

## 1 Introduction

Zeolitic imidazolate frameworks (ZIFs), a subfamily of metal‐organic frameworks (MOFs), exhibit intricate structures with nano‐sized pores. These frameworks, composed of transition metal ions and imidazolate linkers, play a pivotal role in various applications.[^1^, ^2^] They have been extensively studied for their potential applications in various fields, including gas storage, separation, and catalysis.[^3^, ^4^] Among them, ZIF‐8 and ZIF‐67 have garnered significant attention due to their exceptional properties, including strong thermal and chemical stability, high internal surface area that allows for efficient gas adsorption, and tunable pore size that can be adjusted by selecting appropriate ligands for improved selective CO_2_ adsorption, for example.[^5^, ^6^, ^7^] ZIF‐8 is promising for producing syngas, a mixture of carbon monoxide and hydrogen, through CO_2_ reductive reaction (CO_2_RR) owing to its high surface area, well‐defined pore structure, and ligand properties.^[8]^ The importance of syngas lies in its industrial application, serving as a crucial fuel mixture in various industrial processes, spanning from chemical synthesis to electricity generation. However, sustainable syngas production remains challenging, and CO_2_RR offers a promising route,[^9^, ^10^] especially considering the need to mitigate greenhouse gas emissions.^[11]^

The synthesis of ZIFs is performed using various methods, including hydrothermal,^[12]^ room‐temperature,^[13]^ sonochemical,^[14]^ mechanochemical,^[15]^ microwave‐assisted,^[16]^ and ionothermal synthesis.[^17^, ^18^] The ZIF‐8 porous framework integrates imidazole ligands undergoing tetrahedral coordination with zinc ions (Zn^2+^). ZIF‐67 contains cobalt (Co) instead of Zn, giving rise to a Co (II)‐imidazole framework.^[19]^ The traditional methods for synthesizing ZIF‐8 and ZIF‐67 involve the use of expensive and toxic organic solvents such as N, N‐dimethylformamide (DMF), often difficult to remove from the pores of the crystals.

This study focuses on an environmentally friendly synthesis approach using a steam‐assisted dry gel method to optimize DMF‐free ZIFs for CO_2_RR. In this environmentally friendly approach, we use water vapor as a solvent to form a gel‐like precursor within an autoclave, eliminating the use of hazardous organic solvents. The dry gel method is advantageous because it allows for synthesizing highly porous ZIFs with a high degree of control over the particle size and morphology.[^20^, ^21^] The autoclave serves as the controlled environment for ZIF crystallization. In general, the topology of ZIFs is influenced by several factors, including the source of the metal center (zinc or cobalt in this case), the ratio of imidazole molecules to metal salts, the amount of water used in the synthesis, among others.

In this work, by systematically varying the volume of water vapor during synthesis, we achieve two critical objectives: controlling the particle size and increasing the homogeneous particle distribution of ZIFs. As a result, we unlock the latent potential for enhanced syngas production. The key lies in how water content influences ZIF‐8’s structure, textural properties, and electrochemical performance. By comparing the CO_2_RR performance of ZIF‐8 and ZIF‐67 (made with the same linker), we provide new insight into the crucial role of the metal center of MOFs in driving this reaction.

## Experimental

### Materials

Zinc acetate dihydrate (Zn(OAc)_2_.2H_2_O, Sigma‐Aldrich, ≥98 %), Cobalt (II) acetate tetrahydrate (Co(OAc)_2_.4H_2_O, Sigma‐Aldrich, ≥98 %), 2‐Methylimidazole (2‐MeIm, Sigma Aldrich, 99 %), Deionized (DI) water and ethanol were used without further purification.

### Preparation Method

ZIF‐8 and ZIF‐67 were synthesized via a steam‐assisted synthesis method using a 100 mL Teflon‐lined stainless‐steel autoclave, as illustrated in Figure S1. Zinc and cobalt acetates were the precursors for ZIF‐8 and ZIF‐67, respectively, and 2‐methylimidazole was used as the linker. Typically, a mixture of 2.5 mmol of a metal salt and 25 mmol of 2‐methylimidazole was poured into a 30 mL quartz ceramic crucible. The crucible was fixed in the middle of the autoclave with a specific volume of water (2, 10, 20 and 30 mL) at the bottom. The autoclave was then closed and transferred into an oven set at 120 °C for 3 hours. After cooling to room temperature, the autoclave was opened, and the precipitate was separated by centrifuge at 4500 rpm, washed three times with a mixture of water and ethanol in a volume ratio of 2 : 1. The powder was dried under vacuum at 60 °C, and the weight of the powder obtained was 0.368 g. The samples are named ZIF‐8_X or ZIF‐67_X, with X being the volume of water used during the synthesis. For example, ZIF‐8_20 means ZIF‐8 synthesized with 20 mL of water.

### Preparation of Gas Diffusion Electrode (GDE)

7.5 mg of ZIF‐8 or ZIF‐67 was mixed with 450 μL Isopropanol and 40 μL Nafion® 117 solution (5 wt %) by sonication for 30 minutes. The slurry was drop cast on a 1 cm×1.5 cm gas diffusion layer (SIGRACET GDL 28BC, Ion Power GmbH). The GDL 28BC is a proven substrate of the gas diffusion electrodes (GDEs) for energy applications.[^22^, ^23^, ^24^] The obtained GDEs were left to dry overnight at room temperature. The catalyst loading is about 1.6 mg cm^−2^.

### Physicochemical Characterization

The X‐ray Powder Diffraction (XRD) was collected to verify the sample crystallinity. XRD data were acquired using a Rigaku Geigerflex D Max‐C Series diffractometer employing Cu−Kα radiation, scanning in 0.02° 2*θ* increments, with a count time of 197 seconds per step.

The as‐prepared ZIF‐8 and ZIF‐67 catalyst morphology was studied by Field Emission Scanning Electron Microscopy (FESEM, Supra40 from Carl Zeiss) at 5 kV. The adsorption‐desorption isotherms of N_2_ and CO_2_ at −196 and 25 °C, respectively, were recorded using a Microtrac Belsorp MAX II HP. Before each experiment, the samples were degassed under vacuum at 150 °C for 3 hours using a heating rate of 5 °C min^−1^. The specific surface area was determined by applying the Brunauer–Emmett–Teller (BET) theory to the −196 °C N_2_ adsorption data (with an R^2^=0.999), while the pore volume and pore size distribution curves were obtained by applying the non‐local density functional theory (NLDFT) model. The CO_2_ adsorption isotherm was subsequently performed after the N_2_ adsorption‐desorption isotherm.

### Electrochemical Tests

A custom‐made three‐compartment flow cell (Figure S2) was utilized to evaluate the catalytic CO_2_RR in 2 M KHCO_3_ and 1 M KOH electrolytes.^[25]^ An iridium‐coated titanium plate (Ir‐MMO) and a mini Ag/AgCl (1 mm, leak‐free LF‐1) were used as the anode and reference electrodes, respectively. The reference electrode is immersed in the catholyte and separated from the anolyte by the membrane. The GDE separates the catholyte from the gas compartment, with the catalyst facing the catholyte. When KHCO_3_ electrolyte was used, a Nafion membrane (N117, Ion Power) was utilized, avoiding the re‐oxidation of CO_2_RR products. Both catholyte and anolyte were KHCO_3_ solution and circulated through the compartments at 4 mL min^−1^ during the test, using a peristaltic pump. The anolyte was saturated with CO_2_ gas at a constant 9 mL min^−1^ flow. A constant CO_2_ flow of 25 mL min^−1^ was injected in the gas compartment, which diffused through the GDE to reach the catalyst. Bruker mass flow controllers were used to control the CO_2_ flows.

When KOH electrolyte was used, an anion exchange membrane (Sustainion® 37–50, Dioxide materials) was employed. The KOH anolyte was recirculated, while the KOH catholyte was supplied in a single‐pass mode. The flow rate for both anolyte and catholyte was 4 mL min^−1^.

Electrochemical tests were performed using a CHI760D potentiostat. Each chronopotentiometry test was run for at least 30 minutes after an iR compensation of 85 %.

Electrode potentials were then calculated vs. RHE reference by applying the Nernst equation,
(1)

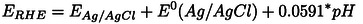




Where *E*_Ag/AgCl_ is the applied potential measured by the Ag/AgCl reference electrode, $E^{0}\left(Ag/AgCl\right)$ is the potential of the reference electrode versus the Standard Hydrogen Electrode (SHE).

The stability test was performed in the zero gap cell (Figure S2c) on an electrode with the same mass loading (1.6 mg cm^−2^). The anolyte was 0.5 M KHCO_3_ and recirculated with the help of a peristaltic pump. The CO_2_ was sent to the cathode compartment, and a Sustainion membrane was sandwiched between the cathode and IrO_2_‐coated Ti felt (anode). The test was performed at a fixed current of −300 mA, translating to a current density of −60 mA cm^−2^.

Gas‐phase products were analyzed online by a micro gas chromatograph (μGC, Fusion, INFICON), composed of two channels with a 10 m Rt‐Molsieve 5 A column and an 8 m Rt−Q‐Bond column, respectively. Each channel has a microthermal conductivity detector. The inlet of μGC was connected to the cathodic side of the electrochemical cell.

High‐performance liquid chromatography (HPLC) (Shimadzu Prominence HPLC) was used to analyze the liquid products, which was equipped with a ReproGel column and an ultraviolet‐visible (UV‐Vis) detector set at 210 nm, with a mobile phase of 9.0 mM H_2_SO_4_ and a 1.0 mL min^−1^ flow rate.

The FE was calculated using the following Equation (2), which involves dividing the amount of charge required to produce a specific number of moles (N) of a product by the total charge consumed during the corresponding reduction period (Q),

(2) $$FE=\frac{nNF}{Q}\;$$

where n is the mole of electrons required to obtain one mole of this product (n=2 for CO, HCOO^−^ or HCOOH and H_2_; n=12 for C_2_H_4_ and C_2_H_5_OH; n=8 for CH_4_ and CH_3_COO^−^ or CH_3_COOH) and F is the faraday constant.

## 2 Results and Discussion

**Figure 1:**
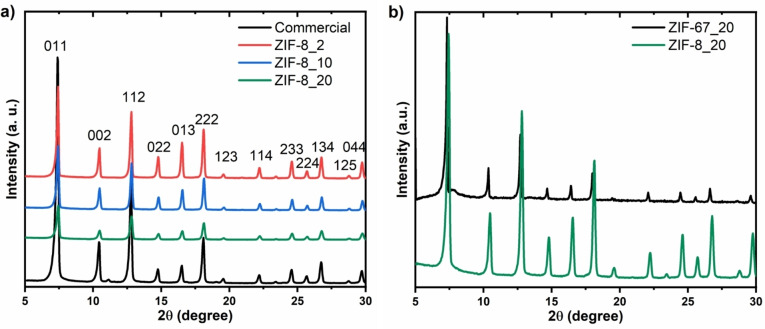
XRD pattern of (a) ZIF‐8 synthesized with different volumes of water and its commercial analogue, (b) ZIF‐8 and ZIF‐67 synthesized with 20 mL of water.

Powder XRD was used to examine the crystallinity and phase purity of the ZIF samples. Figure 1 shows the XRD patterns of the ZIF‐8, ZIF‐67 and commercial ZIF‐8 as reference. Figure 1a compares the diffraction patterns of ZIF‐8 synthesized with different volumes of water to that of commercial analogue. The results show that all samples have the same Bragg peaks as the commercial analogue, indicating that they are single‐phase ZIF‐8 material.

The phase is indexed in cubic symmetry and in the $I\bar{4}$ I3 *m* space group. The peak intensity can give more insights into the crystallinity of these samples as it increases with the crystallinity. For ZIF‐8 samples, the peak intensity increases with the increase in water volume, asserting the importance of controlling the water volume in synthesizing high crystallite ZIF‐8. Figure 1b compares the XRD pattern of ZIF‐8_20 and ZIF‐67_20 synthesized with the same water volume. Since ZIF‐67 is isostructural to ZIF‐8, they share the same standard XRD pattern. Furthermore, based on the Scherrer theory,^[26]^ the peak broadness is inversely proportional to the crystal size of the material. In this study, the XRD peaks of ZIF‐8_20 are broader than those of ZIF‐67_20, hinting at smaller ZIF‐8_20 particle sizes compared to ZIF‐67_20, evidencing the effect of the salt source on the ZIF crystal size distribution.

**Figure 2:**
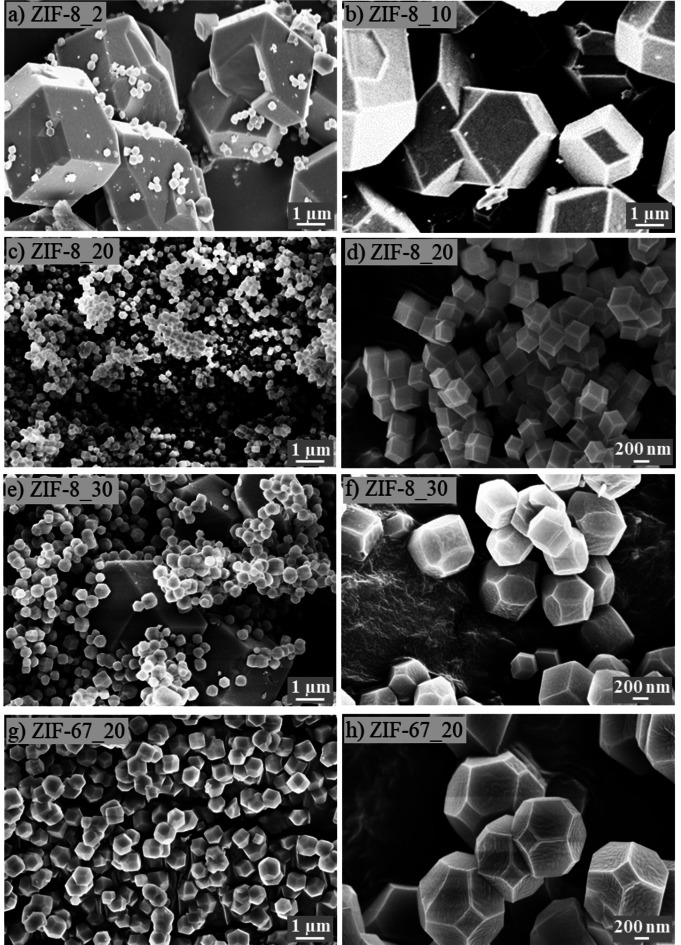
FESEM images of a) ZIF‐8_2, b) ZIF‐8_10, c) and d) ZIF‐8_20, e) and f) ZIF‐8_30, g) and h) ZIF‐67_20.

The physical and textural properties of the biochar sorbents were studied by FESEM and −196 °C N_2_ adsorption‐desorption isotherms. Figure 2 shows the FESEM images of the ZIF‐8_X and that of ZIF‐67_20. For ZIF‐8_2, inhomogeneous crystals with an average size of 2 μm are observed. Many smaller particles are also displayed over the bigger crystals, probably due to unreacted salts resulting from the incomplete reaction owing to water unavailability (Figure 2a). As the water content increases to 10 mL, these smaller particles disappear, accompanied by the decrease in inhomogeneity of a new truncated cubic structure (Figure 2b). Further increase in the volume of water to 20 mL transforms the particles into rhombic dodecahedron crystals, which are visible in Figure 2c and d. A further increase in water content to 30 mL is detrimental to the homogeneity of the particles (Figure 2e), which are composed of big rhombic dodecahedron crystallites sparsely dispersed between smaller truncated rhombic dodecahedron particles (Figure 2f). The availability of more water vapor could accelerate the coalescence of smaller crystallites into bigger ones. This shows again the importance of monitoring the water content during dry‐gel synthesis in achieving uniform particle distribution. For more evidence, the size distribution was estimated for each sample and was reported in Figure S3. The size distribution shows the smallest distribution for ZIF‐8_20, supporting the XRD results.

The morphology of ZIF‐67_20 (Figure 2g and h) shows distinct properties with respect to that of the ZIF‐8_20 (Figure 2c and d) in terms of both the size and shape, using the same content of water in the synthesis, emphasizing again the importance of the nature of salt in tuning the morphology of ZIF materials. It again confirms the XRD results showing smaller particle sizes for ZIF‐8_20.

**Figure 3:**
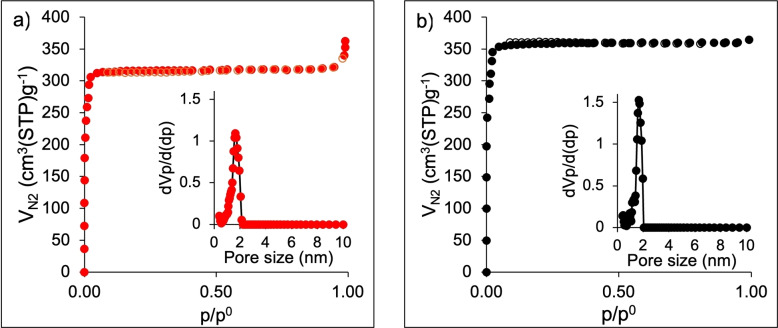
196 N_2_ adsorption‐desorption isotherms for a) ZIF‐8_20 and b) ZIF‐67_20 materials. Closed symbols correspond to the adsorption process, whereas empty symbols are associated with the desorption process. The inset shows the pore size distribution curve for the respective sample.

Figure 3 illustrates −196 N_2_ °C adsorption‐desorption isotherms for ZIF‐8_20 and ZIF‐67_20. Both ZIF‐8_20 and ZIF‐67_20 samples demonstrate a Type I isotherm curve, in accordance with the IUPAC classification,^[27]^ commonly observed in microporous materials. The BET specific surface areas for ZIF‐8_20 and ZIF‐67_20 are 1239 and 1408 m^2^ g^−1^, respectively, accompanied by pore volumes of 0.74 and 0.82 cm^3^ g^−1^. The pore size distribution curves reveal a narrow distribution, with the predominant pore size centered around 1.7 nm for both materials. When NLDFT method is applied. The findings align with those reported in the literature.[^28^, ^29^]

**Figure 4:**
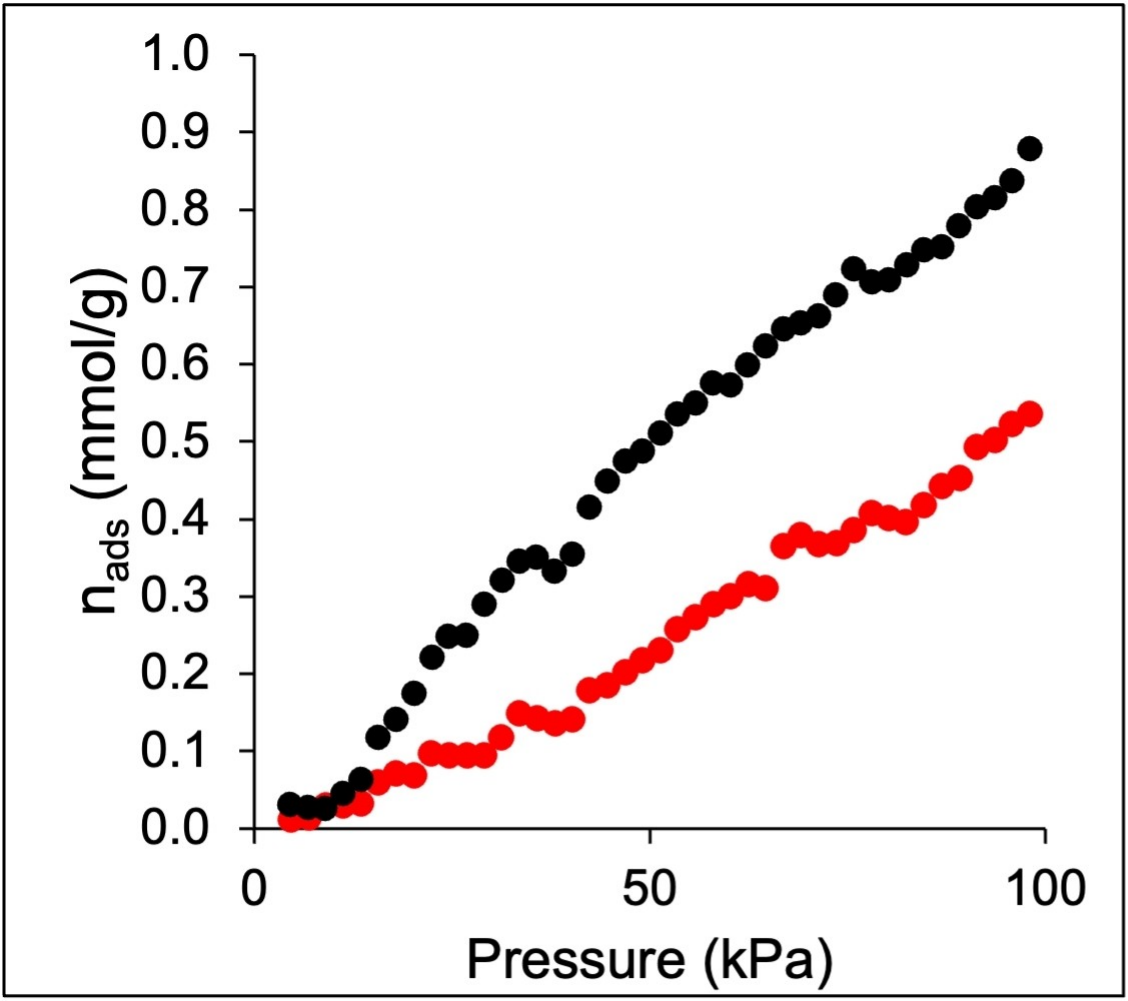
Adsorption isotherms of pure CO_2_ at 25 °C in ZIF‐8_20 (red symbols) and ZIF‐67_20 (black symbols).

Both ZIF‐8 and ZIF‐67 underwent testing in the pure CO_2_ adsorption isotherm at 25 °C (Figure 4) after the −196 °C N_2_ adsorption‐desorption isotherms to understand the CO_2_ affinity of these two materials. It is noted that ZIF‐67 demonstrates an ability to adsorb up to 0.88 mmol g^−1^ of CO_2_, whereas ZIF‐8 exhibits an adsorption capacity of 0.55 mmol g^−1^ for the same gas at 100 kPa. The comparison of N_2_ and CO_2_ adsorption isotherms indicates the existence of more small pores or pore windows between 3.3 Å (kinetic diameter of CO_2_ molecules) and 3.64 Å (kinetic diameter of N_2_ molecules) in ZIF‐67 and thus possibly higher CO_2_ availability for further reduction.

Table S1 summarizes and compares the physical and textural properties of ZIF‐8 and ZIF‐67 synthesized by various methods. This comparative study evidences that our method produces samples with surface area and pore volume amongst the highest. Furthermore, our samples exhibit high CO_2_ adsorption with a particle size distribution comparable to those in the literature. This depicts the excellent properties obtained with this environmentally friendly synthesis process.

**Figure 5:**
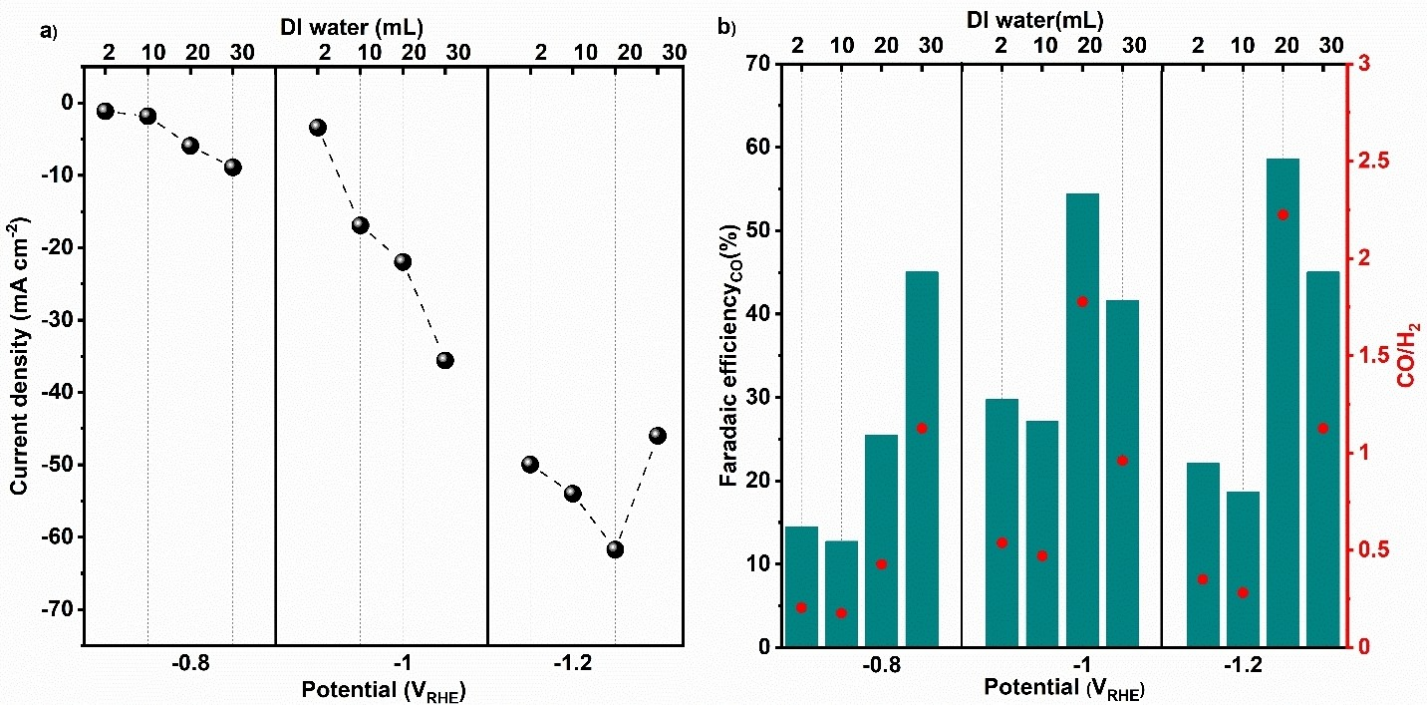
Current densities of ZIF‐8_ (2, 10, 20, 30) electrodes at different potentials (a) and the corresponding CO faradaic efficiency and CO: H_2_ ratio (b) with 2 M KHCO_3_ electrolyte. Dotted lines in (a) provide guides to the eye.

Due to its high porosity and confined metal centre, ZIF can be an excellent catalyst for CO_2_ reductive reaction. In that regard, the catalyst must remain stable under reductive potential with a steady current. Figure S4 shows the chronopotentiometry of different ZIF‐8 electrodes at different potentials in a flow cell configuration using 2 M KHCO_3_. ZIF‐8_20 exhibits the steadiest current density for all potentials within an extended period. At −1.2 V_RHE_, the highest current density of approximately 60 mA cm^−2^ is obtained for the ZIF‐8_20 electrode (Figure 5a). The relatively lower current compared to other metal catalysts is due to the inherent insulating nature of these MOFs.

However, this material proves to be a good candidate for syngas production with different ratios of CO: H_2,_ as shown in Figure 5b, displaying the Faradaic efficiency (FE) of CO of the ZIF‐8 electrodes in 2 M KHCO_3_ and under different reductive potentials. All ZIF‐8 electrodes produce formate with FEs in the 10–15 % range. The samples synthesized with low water contents, ZIF‐8_2 and ZIF‐8_10, depict the lowest FE_CO_ for all potentials from −0.8 V_RHE_ to −1.2 V_RHE_. The FE_CO_ increases as the water content increases during the synthesis, and ZIF‐8_20 shows optimum FE_CO_ values of 55–59 % from −1.0 V_RHE_ to −1.2 V_RHE_. Further raising the water content to 30 mL in the synthesis, the ZIF‐8_30 exibits steady FE_CO_ of about 45 % at all investigated potentials.

The CO selectivity of the catalysts changes by altering their synthesis conditions, which is advantageous for the production of syngas with different CO: H_2_ ratios. The ZIF‐8_2 and ZIF‐8_10 electrodes produce syngas with low CO: H_2_ ratios (<1) at all investigated potentials, while the ZIF‐8_20 favors the production of syngas with a CO: H_2_ ratio around 2. A further increase in the water content during synthesis results in ZIF‐8_30 sample able to produce syngas with a CO: H_2_ ratio near 1. This characteristic holds significant promise for advancing the widespread adoption of syngas as a clean alternative to fossil fuels, with the potential to yield substantial benefits in both electricity generation and liquid fuel production.

**Figure 6:**
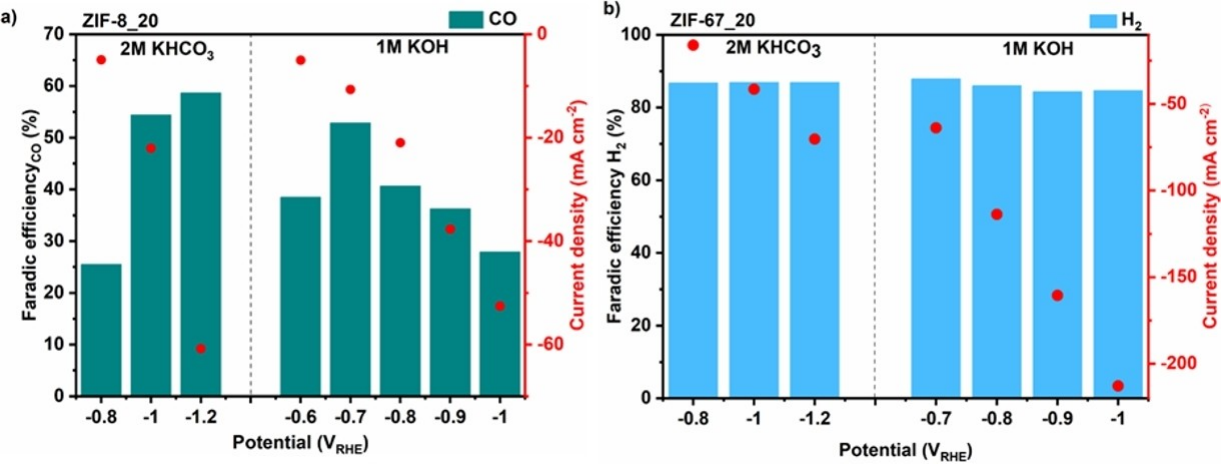
Faradaic efficiency for CO on ZIF‐8_20 electrodes (a) and faradaic efficiency for H_2_ on ZIF‐67_20 electrodes (b) at different potentials in 2 M KHCO_3_ and 1 M KOH electrolytes.

The high CO_2_RR performance for ZIF‐8_20 can be associated with its small particle size. Such structure exposed more Zn (II) metal center density,^[30]^ which can readily react with the CO_2_ molecule. The nature of the electrolyte also plays a vital role in the performance of CO_2_RR^[31]^ and the tunability of products like syngas. It influences the movement of CO_2_ molecules and the electron transfer needed for the reaction. This creates a unique microenvironment where the reaction occurs at the three‐phase interface (solid electrode, liquid electrolyte, and gas phase).^[32]^ Figure 6 compares the FE of the gaseous products for ZIF‐8_20 and ZIF‐67 in 2 M KHCO_3_ and 1 M KOH_._ ZIF‐8 yields the same gaseous products regardless of the electrolyte but in different proportions (Figure 6a). Instead, all electrodes show a similar formate selectivity of about 3 %, much less than the 10–15 % obtained in KHCO_3_ electrolyte. The predominant products remain CO and H_2_. The CO_2_RR in the alkaline electrolyte is more energy‐efficient since FE_CO_ at −1.2 V_RHE_ in 2 M KHCO_3_ could be readily reached at a lower potential value of −0.7 V_RHE_ in 1 M KOH. The current density is also enhanced in the KOH electrolyte with respect to the KHCO_3_ one at the same potential. This is likely related to the higher ionic conductivity of KOH, which can drive the mass diffusion compared to almost neutral KHCO_3_. The tunability of the CO:H_2_ ratio is less evidenced in KOH with only a high CO:H_2_ of 1.6 at −0.7 V_RHE_, and this ratio remains lower than one at other potentials. Concisely, the FE_CO_ is significant at low overpotentials in the KOH electrolyte. At the same time, it is much enhanced at high overpotentials in KHCO_3_ one, highlighting the vital role of the electrolyte in the CO_2_RR. Hori et al.^[33]^ discovered that the type of electrolyte directly affects the CO_2_ conversion products due to how pH impacts the availability of H^+^ ions on the electrode surface. Considering the pH around the cathode is essential to optimize syngas production since the conversion process uses up H^+^ and produces OH^−^. Besides CO and H_2_, this process also drives formate production in a relatively lower proportion. Despite the benefits mentioned above, KOH has some drawbacks that must be addressed in the long run. Due to the formation of carbonate salt when CO_2_ encounters KOH, high carbon loss is noticeable in the electrolyte. The carbonate formation within the GDL during electrowetting can easily alternate the hydrophobicity of the GDL. This crystal can also block the pores of the GDL, limiting the CO_2_ diffusion to the catalyst layer. Figure 6b shows that ZIF‐67_20 is more favorable for hydrogen evolution reaction (HER) regardless of the potential in 2 M KHCO_3_ and 1 M KOH, with little formate formation observed. This discrepancy in the CO_2_RR of ZIF‐8 and ZIF‐67 can be explained by the difference in their metal centers. Although ZIF‐67 maintains a strong interaction with the CO_2_, as shown in Figure 4, the Co (II) center does not activate the CO_2_ molecules for reduction. Instead, the Zn (II) metal center in ZIF‐8 can activate CO_2_ molecules instead of the H_2_O ones, making the selective electroreduction of the former more likely. This observation highlights the necessary interaction between the metal center and CO_2_ molecule for high‐performance CO_2_RR.

The selectivity of ZIF‐8 and ZIF‐67 towards CO and HER, respectively, has been reported in the literature. Xiaole Jiang et al.^[34]^ found by density function theory (DFT) calculations and XAS measurements that the ligands coordinated with the metal center are the active sites in ZIF‐8 and ZIF‐67 instead of Zn and Co during CO_2_RR. Furthermore, they determined that the nature of the metal center is essential for selectivity since the Zn metal center in the CO_2_RR was favorable for CO production with a FE_CO_ value of 81.0 % at −1.1 V_RHE_, and the ZIF‐67 only producing hydrogen. These results are in agreement with this work. Furthermore, they also confirmed that these MOFs remain stable within the potential range reported. Yulin Wang et al.^[35]^ report is also in line with this work. Comparing ZIF‐8 grown with different Zn salts with ZIF‐67, they also found little to no CO_2_ reduction reaction for ZIF‐67, unlike for ZIF‐8, which shows high CO_2_RR towards CO with a maximum FE_CO_ of 65 %. S. Dou et al.^[36]^ suggested that for ZIF‐8, the activation of CO_2_ molecules can be achieved by an electron‐rich center, which promotes the transfer of electrons from the active sites to the antibonding orbitals of CO_2_, creating more ^*^COOH, a critical intermediate in CO production. Incorporating imidazole‐based ligands into ZIFs can also increase their metal basicity, ease the adsorption and activation of the Lewis acid CO_2_, and enhance their CO_2_ reduction performance.^[37]^ Moreover, the size and shape of the ZIF‐8 particles can affect their selectivity and catalytic activity,[^38^, ^39^] as explained above in Figure 5. Sassone et al.,^[40]^ through the design of a dual‐function electro‐organocatalyst, demonstrated using DFT calculations that the catalyst takes advantage of the imidazolate lone pair's ability to bind CO_2_ and resulting in CO production during CO_2_RR after the organic imidazolate ligand activates the catalyst, underscoring the importance of surface imidazolate as active site for CO_2_‐to‐CO conversion with an excellent FE_CO_ of 70.4 % at a potential of −1.2 V_RHE_. However, this is inconsistent with the present work, which underscores the importance of the metal center instead of the ligand. Even though ZIF‐8 and ZIF‐67 share the same imidazolate ligand, they have shown distinct performance in the CO_2_RR owing to their different metal centers. However, the slight production of formate on ZIF‐67 could hint at a weak CO_2_ activation on the ligands, but this is not the dominant CO_2_ activation step.

We believe the predominant CO_2_ activation step occurs on the metal center, which binds and polarizes the CO_2_ molecules, making them more susceptible to reduction by electrons, in consistence with the literature.^[41]^ The imidazolate ligands, with their nitrogen‐rich atoms, can donate electrons and actively participate in the reaction pathway. The whole CO_2_RR process is based on the metal center‐ligand cooperation, explaining the good performance of ZIF‐8 and the opposite behavior of ZIF‐67 in catalyzing this reaction.

**Figure 7:**
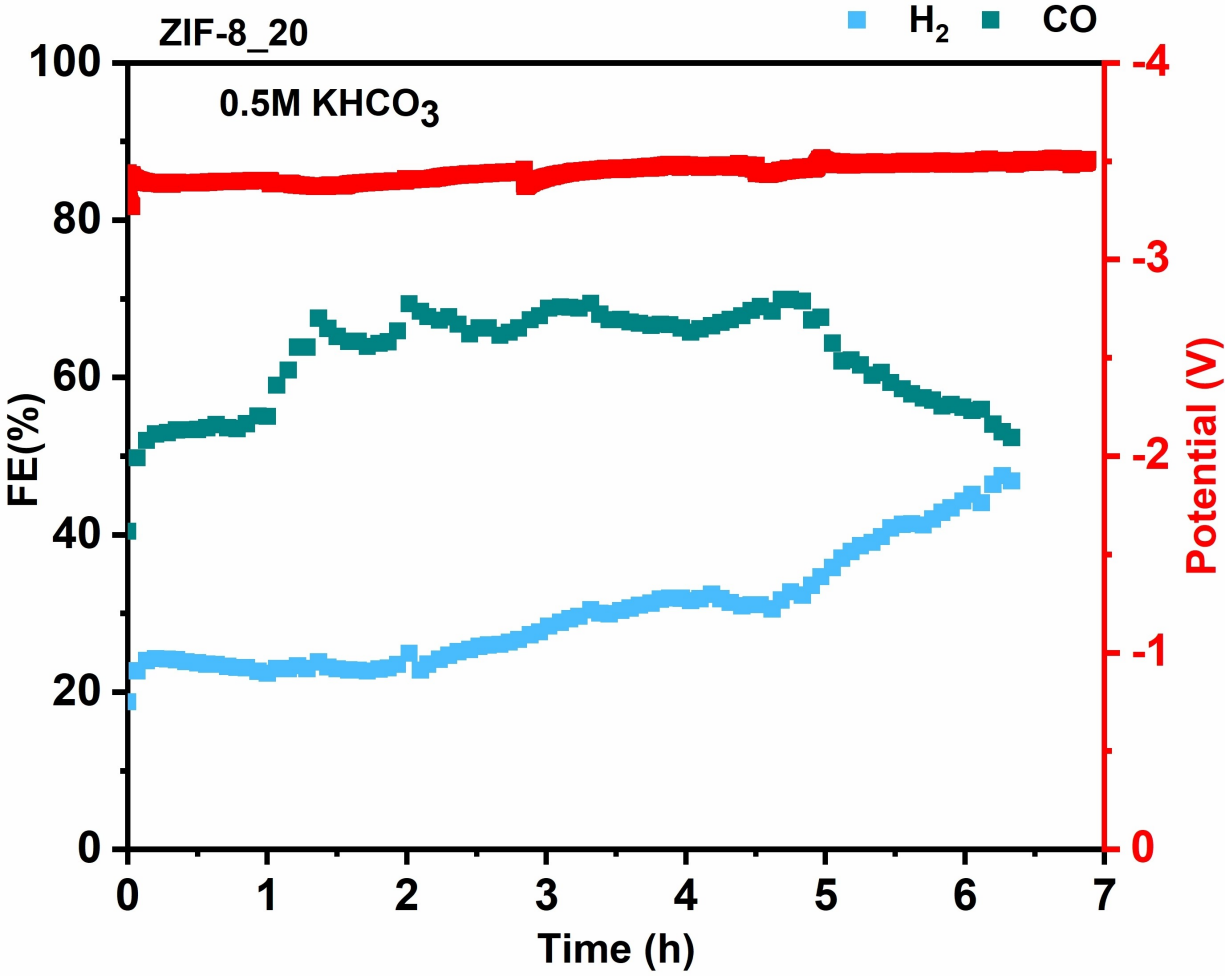
Stability test of ZIF‐8_20 at −60 mA cm^−2^ in 0.5 M KHCO_3_ in MEA cell.

The stability test in the flow cell is unreliable since the GDE wettability is fast due to the short‐lived integrity of the three‐phase solid‐liquid‐gas interface. Therefore, the stability test was performed in the zero gap cell with the same mass loading (1.6 mg cm^−2^), and the result is shown in Figure 7.

It displays that the CO and H_2_ productions stabilized after 2 h of electrolysis with an FE_CO_ of around 60 %, similar to the value reported for the flow cell. However, this value starts to decrease after 5 h, followed by an increase in FE_H2_. This could signal the flooding of the cathode, which is due to various phenomena: the CO_2_RR at the cathode produces water as a byproduct. This water can accumulate within the porous structure of the GDE, leading to flooding. In addition, the pressure difference between the gas and liquid phases can cause the liquid electrolyte to penetrate the GDE. If the liquid pressure exceeds the capillary pressure of the GDE, flooding occurs. The flooding in MEA configuration is still an ongoing research topic, and we are still optimizing our setup.

## 3 Conclusions

In this study, we explored how the shape and morphology of ZIF‐8 materials synthesized via the steam‐assisted dry‐gel technique were influenced by the volume of water used during the synthesis process. It was observed that too low and high water volumes resulted in the formation of heterogeneous structures. A structural homogeneity was achieved only when employing an optimal water volume during synthesis.

The investigation into the efficacy of ZIF‐8 materials as catalysts for CO_2_RR revealed a strong correlation between their structural attributes and performance. The ZIF‐8 catalyst with a more uniform cubic structure and smaller particle size, exposing a higher density of Zn(II) metal centers, achieved superior performance for CO_2_RR with a maximum faradaic efficiency of 59 % for CO formation at −1.2 V_RHE_ in 2 M KHCO_3_ electrolyte.

Contrastingly, with respect to ZIF‐8, the isostructural ZIF‐67 exhibited a slightly higher pore volume and BET surface area, enhancing CO_2_ adsorption, yet displayed limited selectivity towards CO_2_RR. This outcome suggests that factors beyond porosity and CO_2_ adsorption significantly influence CO_2_RR performance. The limited catalytic activity of ZIF‐67 can be attributed to a weak activation of CO_2_ on the Co(II) metal center. Instead, ZIF‐8 features the Zn(II) metal center, which favors the activation of CO_2_ molecules and their subsequent reduction. Moreover, we also posit that the collaborative action of metal centers and ligands within ZIF materials plays a pivotal role in facilitating effective CO_2_RR.

This study provides comprehensive insights into the diverse behaviors exhibited by ZIF materials, offering guidance for the design of novel and efficient ZIF‐based catalysts for CO_2_RR application.

## Conflict of Interests

The authors declare no conflict of interest.

4

## Supporting information

As a service to our authors and readers, this journal provides supporting information supplied by the authors. Such materials are peer reviewed and may be re‐organized for online delivery, but are not copy‐edited or typeset. Technical support issues arising from supporting information (other than missing files) should be addressed to the authors.

Supporting Information

## Data Availability

The data that support the findings of this study are available from the corresponding author upon reasonable request.
