# Supplementary material for: Green Synthesis of MOF‐Based Materials for Electrochemical Reduction of Carbon Dioxide
Source: ChemSusChem. 2024 Oct 4;18(2):e202400684. doi: 10.1002/cssc.202400684 (PMC11739850; doi:10.1002/cssc.202400684)
Supplement: Supplementary file 1 — Supporting Information [file CSSC-18-e202400684-s001.pdf]

ChemSusChem

Supporting Information

Green Synthesis of MOF-Based Materials for Electrochemical Reduction of Carbon Dioxide

Mitra Bagheri, Mirtha A. O. Lourenço, Julien K. Dangbegnon,* Nicolò B. D. Monti, Luís Mafra, Fabrizio Pirri, and Juqin Zeng*

Supporting Information

Green Synthesis of MOF-based Materials for Electrochemical Reduction of Carbon Dioxide

Mitra Bagheri[1,2], Mirtha A. O. Lourenço[3], Julien K. Dangbegnon[1*], Nicolò B. D. Monti[1,2], Luís Mafra[3], Fabrizio Pirri[1,2], Juqin Zeng[1,2*]

[1]Istituto Italiano di Tecnologia – IIT, Centre for Sustainable Future Technologies (CSFT), Via Livorno 60, Turin, 10144, Italy

[2]Department of Applied Science and Technology (DISAT), Politecnico di Torino, Corso Duca degli Abruzzi 24, Turin,10129, Italy

[3]CICECO – Aveiro Institute of Materials, Department of Chemistry, University of Aveiro, 3810-193, Aveiro, Portugal

Corresponding authors: julien.dangbegnon@iit.it; juqin.zeng@polito.it

Keywords: carbon dioxide; electrocatalysis; ZIF; steam-assisted dry gel method, metal center; syngas

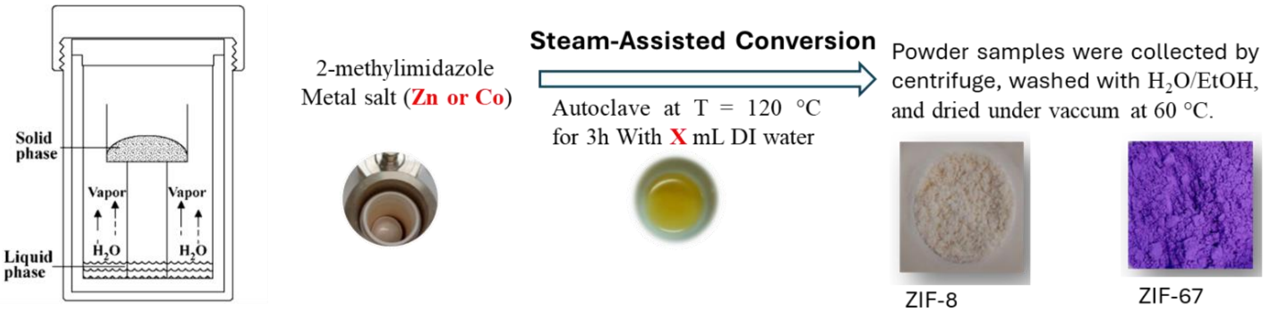

Figure S1: Illustration of the synthesis of ZIFs.[1]

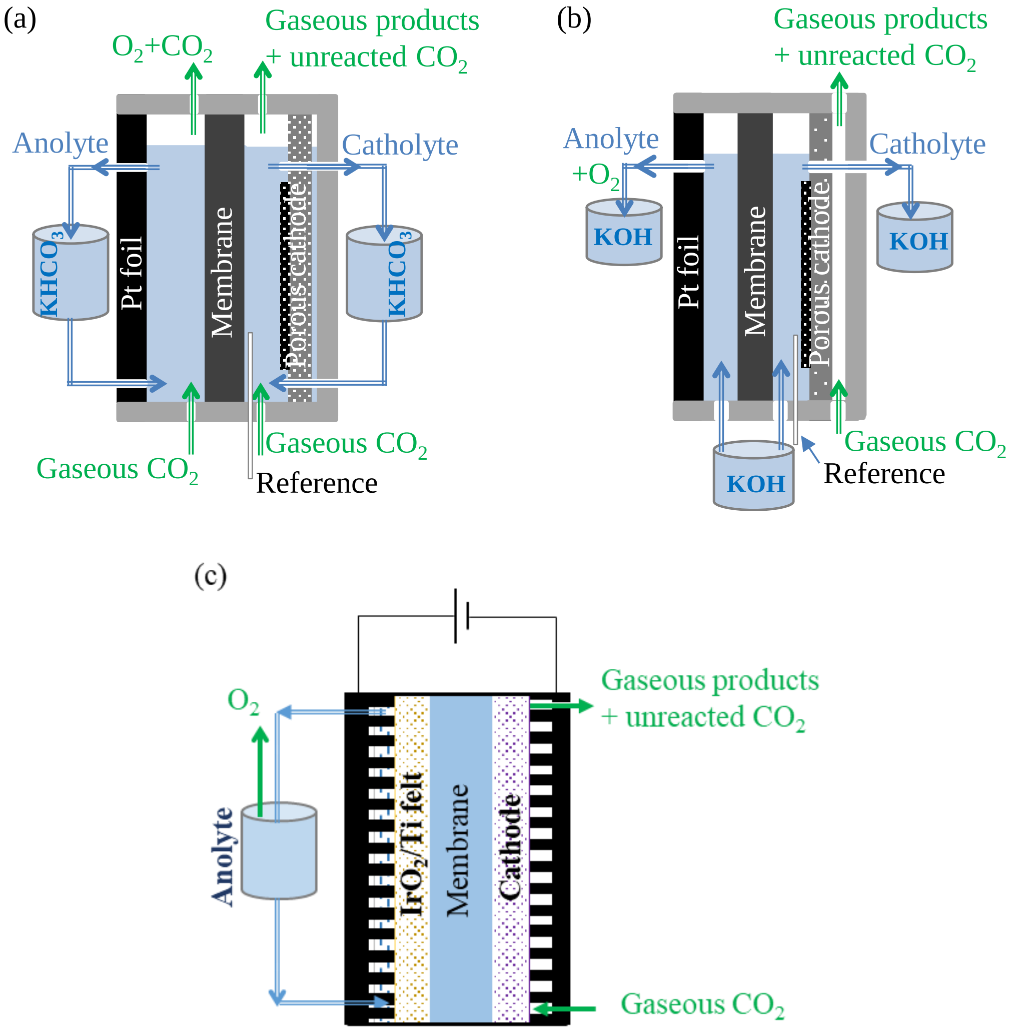

Figure S2: Schemes of the electrochemical cells: (a) three-electrode two-compartment configuration; (b) three-electrode three-compartment configuration; (c) zero-gap cell configuration

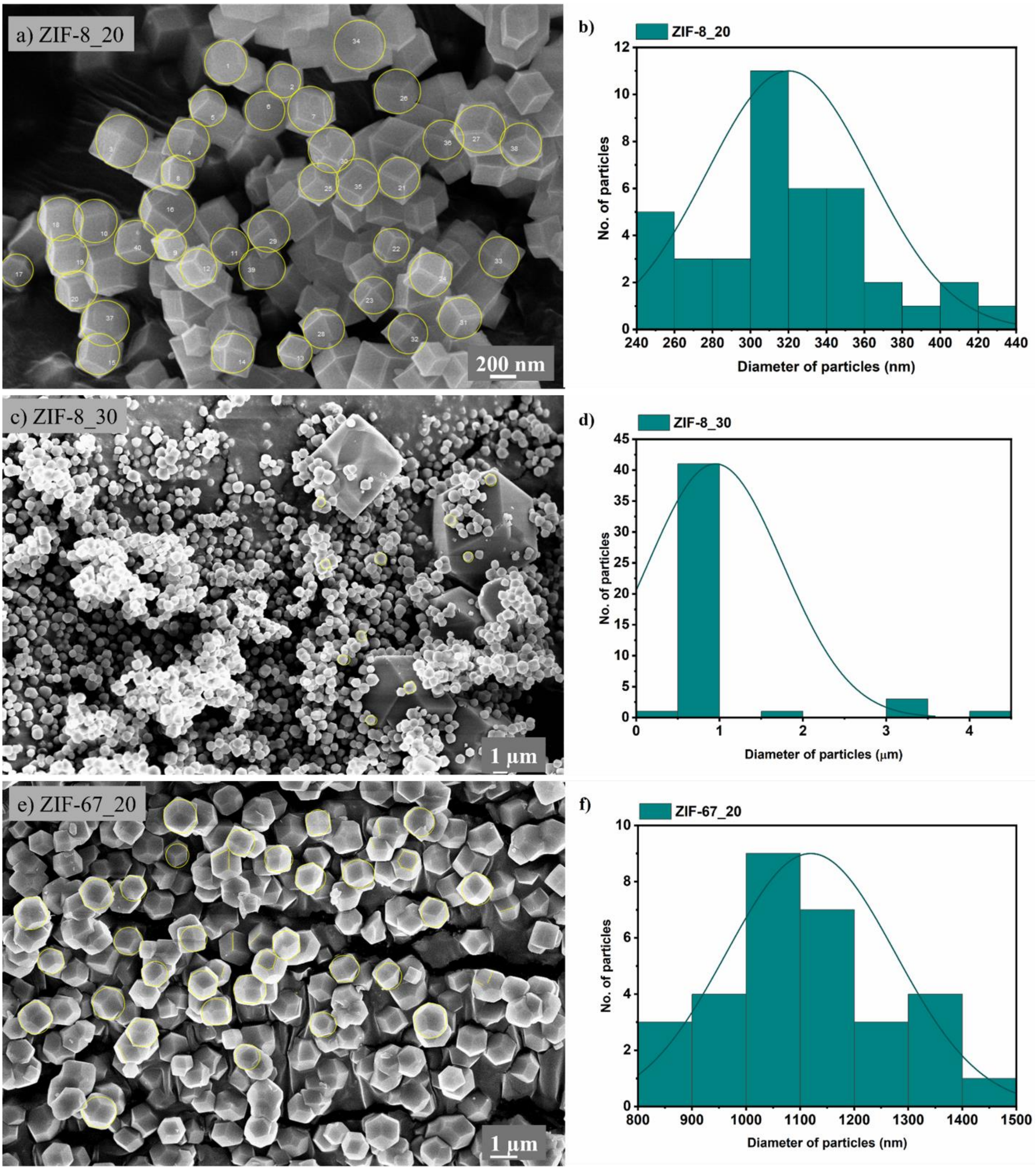

Figure S3: size distribution histogram

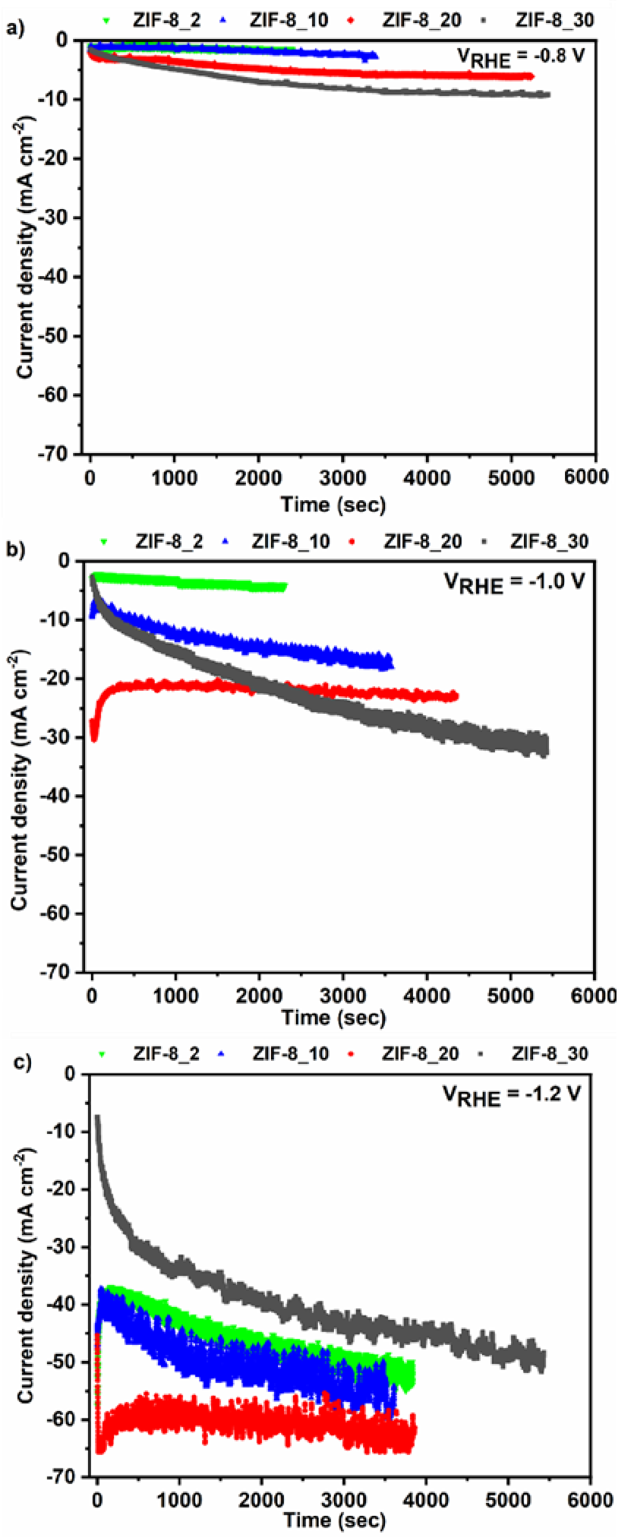

Figure S4: Chronopotentiometry of ZIF-8_ (2,10,20,30) at different V_{RHE}: (a) –0.8 V, (b) –1.0 V, and (c) –1.2 V

Table S1: Comparison of physical and textural properties of ZIF-8 and ZIF-67 synthesized by different methods.

**Materials**	**Synthesis technique and reaction conditions (Solvent, T (°C), t (growth time)**	**Size distribution (nm)**	**BET Surface area (m^2 g^{-1})**	**BET Pore volume (cm^3 g^{-1})**	**BET Pore size (nm)**	**CO_2 adsorption mmol g^{-1}**	**Ref.**
ZIF-8	***Steam-assisted dry gel method (H_2O (v), 120°C, 3h)***	***240-440***	***1239***	***0.74***	***1.7***	***0.55 at 25 °C***	***This work***
ZIF-67	***Steam-assisted dry gel method H_2O (v), 120°C, 3h***	***800-1500***	***1408***	***0.82***	***1.7***	***0.88 at 25 °C***	
ZIF-8	Microwave- Ionic liquid, 140°C, 1h	300—500	471	0.337	-	0.68 at 0 °C	[2]
ZIF-8	Conventional electric heating ionothermal -Ionic liquid, 140°C, 72h	≥500	341	0.207	-	0.45 at 0 °C	
ZIF-8	H_2O, RT, 7 days	150	1356	0.59	1.75	-	[3]
	NH_3, RT, 24h	300-350	1568	0.86	2.19	-	
	MeOH, RT, 24h	200	1333	0.58	1.74	-	
ZIF-8	Microwave-assisted solvothermal-MeOH, 120°C, 30min	215	1573.8	0.69	1.75	0.0995 at 25 °C	[4]
ZIF-8	NH_4OH+H_2O, 120°C, 0.5h	368	1303.9	0.507	1.557	0.1019 at 25 °C	
ZIF-8	reaction‒diffusion framework-DMF, RT, 48 h	500-4000	1302	-	-	-	[5]
ZIF-67	reaction‒diffusion framework-DMF, RT, 48 h	1000-10000	1023	-	-	-	
ZIF-8 1:2 molar Zn/Hmim	Electric current-assisted synthesis MeOH,-, 8 h	stacking of polycrystals	1135	0.48	-	1.39 at 0 °C	[6]
ZIF-8 1:6 molar Zn/Hmim	Electric current-assisted synthesis MeOH,-, 8 h	stacking of polycrystals	1339	0.53	-	1.4 at 0 °C	
ZIF-8 1:6 molar Zn/Hmim	Without Electric current-assisted MeOH, RT, 24 h	200≤	973	0.36	-	0.82 at 0 °C	
ZIF-8	Microwave- H_2O 120°C, 0.5h	190-350	1075	0.49	-	-	[7]

*V stands for vapour

Reference:

[1] Q. Shi, Z. Chen, Z. Song, J. Li, and J. Dong, "Synthesis of ZIF-8 and ZIF-67 by Steam-Assisted Conversion and an Investigation of Their Tribological Behaviors," *Angewandte Chemie International Edition*, vol. 50, no. 3, pp. 672–675, Jan. 2011, doi: 10.1002/anie.201004937.

[2] L. Yang and H. Lu, "Microwave-assisted Ionothermal Synthesis and Characterization of Zeolitic Imidazolate Framework-8," *Chin J Chem*, vol. 30, no. 5, pp. 1040–1044, May 2012, doi: 10.1002/cjoc.201100595.

[3] M. Izadpanah Ostad, M. Niknam Shahrak, and F. Galli, "The influence of different synthetic solvents on photocatalytic activity of ZIF-8 for methanol production from CO2," *Microporous and Mesoporous Materials*, vol. 326, p. 111363, Oct. 2021, doi: 10.1016/j.micromeso.2021.111363.

[4] S. Payra *et al.*, "The structural and surface modification of zeolitic imidazolate frameworks towards reduction of encapsulated CO_2," *New Journal of Chemistry*, vol. 42, no. 23, pp. 19205–19213, 2018, doi: 10.1039/C8NJ04247K.

[5] D. Saliba, M. Ammar, M. Rammal, M. Al-Ghoul, and M. Hmadeh, "Crystal Growth of ZIF-8, ZIF-67, and Their Mixed-Metal Derivatives," *J Am Chem Soc*, vol. 140, no. 5, pp. 1812–1823, Feb. 2018, doi: 10.1021/jacs.7b11589.

[6] Y. Zhou, J. Qiu, M. Ding, X.-F. Zhang, and J. Yao, "Electric current-assisted synthesis of ZIF-8 with stoichiometric metal and ligand precursors for CO2 adsorption," *Journal of Physics and Chemistry of Solids*, vol. 161, p. 110485, Feb. 2022, doi: 10.1016/j.jpcs.2021.110485.

[7] Q. Bao, Y. Lou, T. Xing, and J. Chen, "Rapid synthesis of zeolitic imidazolate framework-8 (ZIF-8) in aqueous solution via microwave irradiation," *Inorg Chem Commun*, vol. 37, pp. 170–173, Nov. 2013, doi: 10.1016/j.inoche.2013.09.061.
